# Unlocking net-zero supply chain performance system through life cycle assessment: Empirical evidence from rubber industry

**DOI:** 10.1016/j.heliyon.2024.e39944

**Published:** 2024-10-30

**Authors:** Alok Yadav, Anish Sachdeva, Rajiv Kumar Garg, Karishma M. Qureshi, Bhavesh G. Mewada, Naif Almakayeel, Mohamed Rafik Noor Mohamed Qureshi

**Affiliations:** a Department of Industrial and Production Engineering, Dr. B. R. Ambedkar National Institute of Technology”, Jalandhar, 144008, India; b Department of Mechanical Engineering, Parul Institute of Technology, Parul University, Waghodia, 391760, India; c Department of Industrial Engineering, College of Engineering, King Khalid University, Saudi Arabia, Abha, 61421, Saudi Arabia

**Keywords:** Supply chain, Net-zero emission, Life cycle assessment, Climate change, Enabler, BWM, Fuzzy-DEMATEL

## Abstract

The ongoing transition from a supply chain (SC) to net-zero emission (NZE) in SC performance must align with Sustainable Development Goal 13 to address climate change using life cycle assessment (LCA). Effective management of LCA significantly impacts an organization's competitiveness. With a lack of technology oversight and its connections with other enabling parameters that increase competitiveness, practices in the manufacturing industry remain limited. This study identifies and evaluates key enablers for LCA adoption in the manufacturing industry to achieve NZE in SC. Based on 123 responses, an exploratory factor analysis (EFA) was employed, followed by the Best-Worst Method (BWM). Further, a Fuzzy-Decision-Making Trial and Evaluation Laboratory (Fuzzy-DEMATEL) was used. The results revealed ‘organizational integration’, ‘data availability and quality, and ‘policy and regulation’ as significant group enablers that support NZSC to compete in the market. It provides insights for industry practitioners and policymakers on optimizing sustainable SC practices. The essential practical recommendations for resource optimization and carbon footprint reduction are also suggested.

## 1 Introduction

The idea of reaching net-zero emissions (NZE) has gained significant support in light of the growing environmental crises and the urgent need to address climate change [1,2]. To reduce its carbon footprint, the global industrial sector must overcome formidable obstacles to switch from carbon-intensive to carbon-neutral operating models. The industrial SC and logistical networks, in particular, are significant sources of carbon emissions.

The carbon footprint of global SC is increased due to its complex structure, which involves multiple businesses located across various nations and continents [3]. Several factors, including long-distance transportation, significant reliance on fossil fuels, energy-intensive production processes, and trash generation, cause emissions within these SCs. By 2050, projections point to a significant rise in global emissions caused by international trade via logistical networks [4].

The amount of emissions in SC varies significantly between industries and geographical areas. Because of their vast networks, industries, including manufacturing, transportation, and agriculture, exhibit very high pollution levels [5]. However, there's a clear global trend among businesses to embrace creative solutions as part of their initiative to drive their supply chains towards carbon neutrality. To achieve NZE within the SC, these strategies involve adopting green technologies, maximizing resource use, and utilizing data-driven innovations [6]. A vital tool for pushing manufacturing supply chains towards NZE, essential for environmental preservation, is LCA, which is one aspect of reaching NZSC [7]. LCA offers essential insights into locations where emissions occur across the SC by thoroughly analyzing a product's or process's environmental effects, from raw material extraction to disposal [8]. With this comprehensive insight, businesses can pinpoint essential opportunities for reducing emissions and make well-informed decisions to lessen their environmental effect.

LCA allows organizations to track their progress toward setting and meeting challenging but attainable emissions reduction targets [9]. Achieving NZE is a shared objective among stakeholders and LCA fosters cooperation by encouraging responsibility and transparency across the supply chain [10]. In general, LCA must be incorporated into the manufacturing supply chain to promote sustainable practices and, ultimately, protect the environment for coming generations. LCA helps manufacturing supply chains reach NZE by assisting them to find ways to reduce emissions in all production areas.

Following an LCA, a garment company decides to source supplies locally and employ more fuel-efficient modes of transportation to reduce transportation-related emissions. Similarly, a food processing business lowers emissions from energy-intensive processes by modernizing machinery and streamlining production schedules [11]. To meet their energy needs, firms also invest in renewable energy sources like solar and wind power. They also consider alternative materials like recycled plastics and sustainable metals to reduce manufacturing-related emissions [12]. Reusing and recycling materials in closed-loop manufacturing systems reduces emissions, as demonstrated by a beverage company's adoption of reusable packaging. These illustrations show how SC eventually led towards NZE and environmental preservation by using LCA to influence strategic decisions to lower carbon footprints [13].

The rubber industry contributes significant environmental impacts and faces challenges in achieving sustainability. Rubber production is resource-intensive, contributing to deforestation, high water consumption, and carbon emissions, particularly in tropical regions where natural rubber is cultivated. The end-of-life disposal of rubber products, such as tires, creates substantial waste, further complicating the industry's environmental footprint [14]. Despite these challenges, the rubber industry offers opportunities for sustainability through innovations in recycling technologies and synthetic alternatives to natural rubber. This sector is subject to increasing regulatory pressure, which can promote the adoption of LCA practices. Given the industry's global scale and environmental significance, this study identifies key enablers for LCA adoption to guide the transition toward NZE in the SC of rubber manufacturing [15].

Despite continued research efforts, there is still a lack of information regarding the establishment of readily comprehensible NZSC [16]. By using the LCA technique, industries are essentially strengthening their capacities in manufacturing, logistics, SC, and recycling procedures [17]. Therefore, to attain NZSC practices in the rubber and tire manufacturing industry, it is imperative to ascertain the elements propelling the implementation of LCA [18,19]. To fill this gap, this study identifies LCA enablers through a literature review and validates them through surveys of academic and industry personnel. The present study aims to answer the following research questions.

RQ1 To identify the key enablers of Life Cycle Assessment adoption in the rubber industry.
RQ2 To rank these key enablers using BWM methods in the context of NZSC.
RQ3 To analyze the causal interrelationships between these enablers using the Fuzzy DEMATEL method.

After reviewing relevant literature, the primary characteristics facilitating LCA adoption were found. These factors were subsequently validated using EFA. 123 responses answers from the Indian rubber sector were used in an empirical study. Validated factors were prioritized according to their importance using the BWM. Furthermore, the Fuzzy-DEMATEL approach was used to determine causal links between the discovered components.

The paper's subsequent sections are organized as follows: Section 2 provides a comprehensive overview of the NZSC techniques and LCA literature. Section 3 discusses the research methodology used in this study. Section 4 has a complete description of the data analysis and results. Section 5 includes discussion. In section 6, the implications of the study are explained. Finally, Section 7 has a conclusion, limitations and future research scope.

## 2 Literature review

**Fig. 1:**
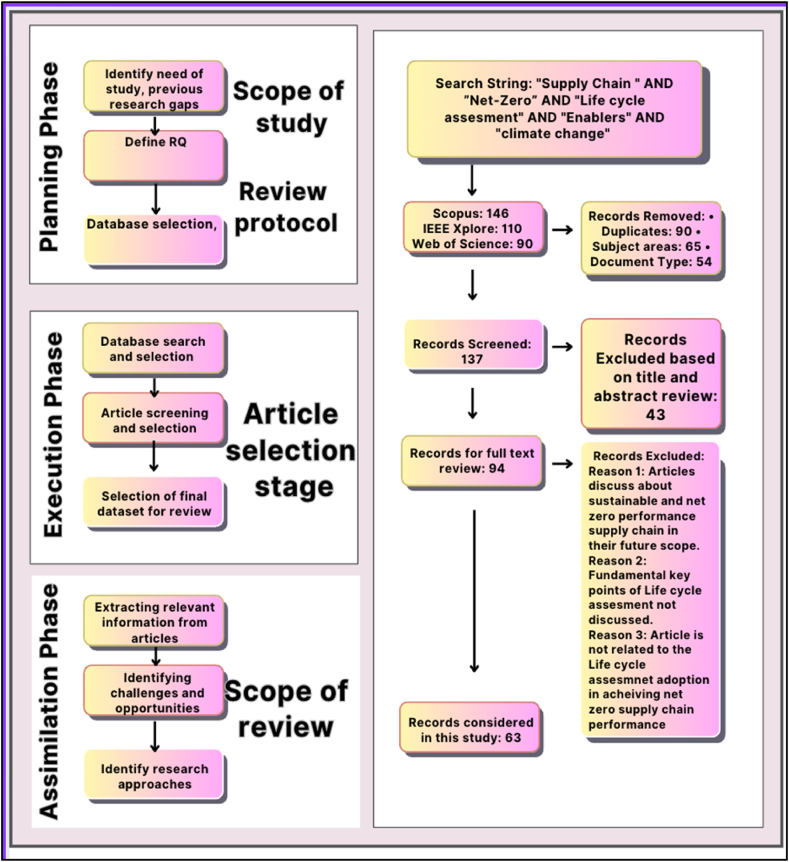
PRISMA for SLR (Authors' work).

This study conducts an SLR to examine how LCA technologies address supply chain difficulties in the rubber industry in the context of achieving NZE. The SLR searched key phrases such as "supply chain," AND "Net-Zero emission," AND "life cycle assessment," AND "climate change," AND "enablers" in major scientific literature databases such as IEEE Xplore, Scopus, and Web of Science. The study used specified inclusion and exclusion criteria, as well as a predefined review methodology, to pick papers. After a careful review of full-text documents, 63 were selected for inclusion. Fig. 1 represents the PRISMA diagram for the SLR process [20].

The United Nations' Sustainable Development Goals (SDGs) identify climate change as one of humanity's most pressing issues caused by excessive human-generated carbon emissions [21]. Zero carbon emission, often known as achieving NZE, is a crucial milestone for organizations today. It is unknown whether achieving NZE is realistic [22]. How might carbon-intensive businesses and supply networks move to carbon neutrality if possible? As the industrial sector transitions to “carbon-neutral” practices and strives for NZE, supply chains play an increasingly important role in these efforts [23]. As a result, reducing the carbon footprint of global SC is critical in the fight for NZE. To reduce the environmental effect of SC, organizations and governments worldwide are rapidly recognizing the necessity of adopting sustainable practices, optimizing logistics, minimizing waste, and transitioning to cleaner energy sources.

### 2.1 Net-zero supply chain

As organizations recognize the need to tackle climate change, there has been an increased emphasis on reaching net zero in supply chains in recent years [24]. The United Nations defines net zero as reducing GHG emissions to the greatest extent practicable, with any residual emissions absorbed by natural processes such as seas and forests. Industrial logistics contribute significantly to emissions at all phases of the SC, including raw material extraction, manufacture, transportation, and disposal [25].

The global SC contributes significantly to GHG emissions, and calculating the exact percentage is difficult. According to studies, these emissions are significant, with global supply chains accounting for up to 68 % of total greenhouse gas emissions [16]. Several international organizations are working to achieve a more carbon-neutral world. For example, the University of California has begun its ambitious "Carbon Neutrality" program, while the Paris government aspires to make the city carbon-neutral by 2050 [24].

Industries like manufacturing aggressively pursue efforts such as the Science Based Targets Project and the Carbon Disclosure Project (CDP) to achieve a net-zero carbon economy [26]. Some businesses are introducing internal carbon pricing schemes to reduce emissions and stimulate investment in greener technologies. Partnerships with suppliers are being developed to incorporate sustainability across the SC, while investments in renewable energy sources are being made to lower carbon footprints even further. Organizations are looking into new technologies like carbon capture and storage (CCS) and sustainable materials to reduce emissions and improve their environmental effect [27].

A NZSC seeks to reduce or compensate for GHG emissions throughout the whole SC network, which includes production, transportation, and disposal [28]. This requires measuring, lowering, and offsetting emissions across the SC. Improving energy efficiency, moving to low-carbon modes of transportation such as electric vehicles, partnering with suppliers on environmental efforts, and using renewable energy sources such as solar or wind power are all ways to reduce emissions. Collaboration amongst stakeholders is critical for promoting sustainable practices and lowering emissions. Implementing an LCA strategy is one of the promising approaches for organizations to systematically identify and address emission reduction possibilities throughout the SC, ultimately leading to NZE [29].

### 2.2 Life cycle assessment and net-zero supply chain

**Fig. 2:**
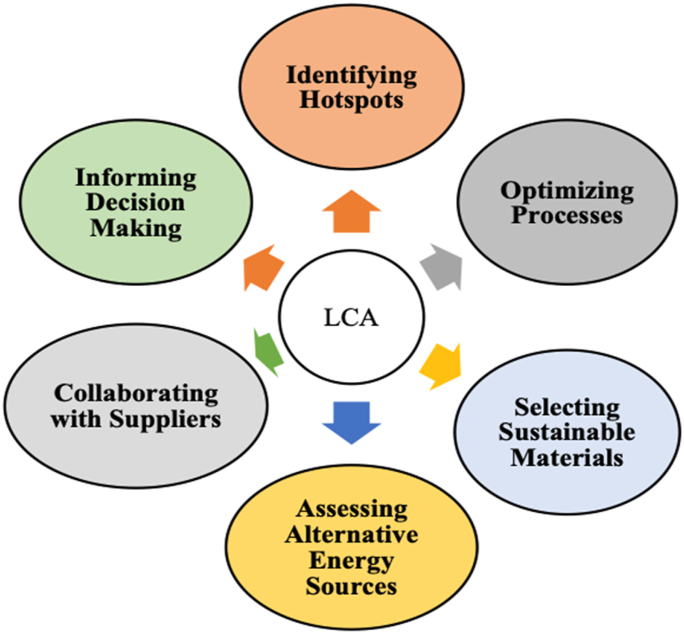
Benefits of LCA to achieve Net-Zero (Authors' work).

The significance of addressing climate change has raised awareness of the need for new solutions to transition to a Net-Zero economy [30]. Organizations increasingly use LCA strategies to reduce emissions and promote environmentally responsible operations. LCA, which assesses a product's environmental impact over its life cycle, has gained popularity as a possible route to achieve net-zero emissions [31]. Unlike conventional approaches focusing primarily on technology, LCA takes a broader approach, evaluating environmental implications at each step of a product's life [32]. When integrated with sustainability, LCA creates a robust framework known as sustainable innovations, which has the potential to address environmental concerns and reach Net-Zero emissions by optimizing resource utilization. Integrating LCA, sustainability, and digital innovation provides a path to decarbonizing the value chain, as shown in Fig. 2, showing the benefits of life cycle assessment in attaining a NZSC. Achieving an NZSC can be significantly aided by applying the LCA technique, which offers a thorough understanding of the environmental impact of products at every stage of their life cycle, from the extraction of raw materials to the disposal of end-of-life materials [18,33]. It includes the following.

(a) *Finding Hotspots:* The supply chain's highest concentrations of greenhouse gas emissions can be found using LCA. Organizations can focus on reducing emissions where they will have the most significant impact by identifying these hotspots [18].
(b) *Optimization of Processes:* Organizations can use LCA to examine every phase of the SC and find ways to cut emissions and increase efficiency. Reducing the environmental impact may entail optimizing packing materials, transportation routes, or manufacturing procedures [34].
(c) *Selecting Sustainable Materials:* Businesses can assess the environmental impact of various industrial materials through LCA. Organizations may cut emissions through the SC by selecting materials with fewer carbon footprints, including recycled or renewable resources [35].
(d) *Assessing Alternative Energy Sources:* LCA can help evaluate the environmental benefits of using renewable energy sources, such as solar or wind power, at various SC stages. Businesses that convert to clean energy can reduce their carbon footprint significantly [36].
(e) *Collaborating with Suppliers:* To evaluate the environmental impact of suppliers' operations and goods, LCA promotes cooperation with them. Organizations may move closer to an NZSC by cooperating to adopt sustainable practices and lower emissions throughout the SC [37].
(f) *Informed Decision Making:* Regarding product design, sourcing, and logistics, LCA offers valuable information and insights to guide decision-making. Organizations can make more sustainable decisions and lead to an NZSC by integrating environmental factors into their decision-making process [38].

Many studies and industries are adopting zero-emission methods, which use creative ways to reduce GHG emissions and mitigate environmental hazards. Table 1 provides a summary of some significant studies. Organizations must change their thinking to attain net-zero operations inside their SC. On the other hand, new assessments highlight how urgently research on SC with zero emissions is needed [39]. The most recent study on the net-zero approach takes a broad perspective, looking at national, regional, and international issues, emphasizing technology and policy. The need for quantitative studies conducted in an industrial environment was stressed to get NZE.

**Table 1:** Summary of studies conducted in the area of LCA and NZSC.

Author	LCA	Net-Zero	SC	Industry	Method	Title
[40]	✓	✓	✕	Buildings	Case study	“Energy life-cycle approach in Net-Zero energy buildings balance: Operation and embodied energy of an Italian case study”
[41]	✓	✓	✓	Buildings	LR	“A review on Life Cycle Assessment, Life Cycle Energy Assessment and Life Cycle Carbon Emissions Assessment on buildings”
[42]	✓	✓	✓	Electric vehicles (EV)	Survey	“Towards a circular and low-carbon economy: Insights from the transitioning to electric vehicles and Net-Zero economies”
[43]	✓	✓	✕	✕	LR	“Applying environmental sustainability boundaries for climate change in life cycle assessment: A review of approaches and implications for policymaking”
[44]	✓	✓	✓	✕	LR	“Net-Zero supply chain performance and industry 4.0 technologies: Past review and present introspective analysis for future research directions”
[45]	✓	✓	✓	✕	LR	“Environmental evaluation of metals and minerals production based on a life cycle assessment approach: A systematic review”
[33]	✓	✓	✓	✕	LR	“Net-zero economy research in the field of supply chain management: a systematic literature review and future research agenda”
[11]	✓	✓	✓	✕	LR	“Drivers, barriers and practices of Net-Zero economy: An exploratory knowledge-based supply chain multi-stakeholder perspective framework”
[46]	✓	✓	✕	✕	LR	“Application of novel technologies to reach net-zero greenhouse gas emissions in the fresh pasteurized milk supply chain: A review”
[47]	✓	✓	✓	Automobile	Survey	“Analyzing the adoption barriers of low-carbon operations: A step forward for achieving net-zero emissions”

### 2.3 Enablers of LCA to achieve NZSC in the rubber industry

Ensuring a NZSC necessitates thoroughly comprehending the environmental effects at every phase of the product life cycle. LCA becomes an essential tool to accomplish this [40]. Carbon management strategies significantly impact three major LCA operating areas related to product, manufacturing and SC. Firstly, LCA allows businesses to create goods with the least possible environmental impact at every stage of their life cycle, from extraction and production of raw materials to usage and disposal. Informed choices about material choice, resource efficiency, and the possibility of implementing circular economy methods are made possible [46]. Secondly, finding environmental hotspots during the production process is made more accessible with the help of LCA. Businesses may optimize energy use, waste output, and emissions by focusing improvement efforts on the areas that have the most influence [47]. Thirdly, LCA encourages collaboration and transparency in the supply chain. Businesses can evaluate the environmental impact of their suppliers and find potential for cooperative efforts towards net-zero goals by using LCA throughout the supply chain [48]. This encourages cooperation among suppliers to put sustainable practices into place and lessen the total impact of the life cycle.

A practical LCA approach enables firms to map their supply chain's environmental footprint, identify areas for improvement, and eventually reach net-zero status. This study aims to identify the enablers that assist the adoption of LCA in the rubber and tire manufacturing industry. In the next part, we will describe how we conducted a systematic literature study to build a comprehensive list of enablers that facilitate LCA adoption [16]. Table 2 shows the enablers found in earlier studies as being connected to LCA implementation.

**Table 2:** Enablers of LCA for NZSC.

Serial No.	Enabler Name	Description	References
1	Stakeholder Engagement	Stakeholder engagement throughout the LCA process makes sure numerous viewpoints are represented.	[1,[43], [44], [45]]
2	Technological Advancements	Technological advancements improve data-gathering methods, modelling methodologies, and analytical tools.	[[46], [47], [48], [49]]
3	Standardization of Methodologies	Standardized techniques improve the ability to compare and reliability among various evaluations.	[20,50,51]
4	Training and Capacity Building	Training programmes develop the abilities required to conduct successful life cycle evaluations.	[27,52,53]
5	Transparency and Disclosure	Transparency of data sources and techniques fosters confidence and dependability in LCA outcomes.	[54,55]
6	Regulatory Support	Supporting rules encourage enterprises to undertake LCAs and reduce their environmental effect.	[40,56,57]
7	Collaboration Across Supply Chain	Collaboration between supply chain stakeholders enables the sharing of information and comprehensive evaluations.	[[58], [59], [60]]
8	Accessibility of Tools and Resources	Relevant resources and instruments democratise LCA, making it more appealing to a larger range of users.	[61,62]
9	Industry Standards and Best Practices	Compliance with industry standards and best practices assures the consistency and integrity of LCA results.	[61,63]
10	Financial Investment	Sufficient financial investment is required to undertake complete life cycle assessments.	[64,65]
11	Policy Alignment	Integration of LCA aims with wider policy goals increases their relevance and impact.	[25,66]
12	Life Cycle Thinking Integration	The incorporation of life cycle thinking into organisational culture ensures sustainability concerns.	[1,67,68]
13	Use of Environmental Management Systems	The use of EMS facilitates the methodical integration of LCA results into organisational procedures.	[11,[69], [70], [71]]
14	Continuous Improvement Culture	Developing a culture of continuous improvement promotes the continued refinement of LCA approaches.	[72,73]
15	Public Awareness and Education	Increasing awareness among people and training stakeholders about LCA increases its acceptance and effect.	[33,74]
16	Incentives for Sustainability Reporting	Offering incentives for sustainability reporting encourages businesses to do LCAs.	[11,75]
17	Flexibility in Methodological Approaches	Flexibility in methodological techniques enables adaption to a wide range of product and industry instances.	[75,76]
18	Government Support and Funding	Government initiatives motivate and subsidise LCA methodology research and development.	[60,76]
19	Supplier Performance Metrics Integration	Integrating LCA data into supplier performance measures encourages sustainable behaviour.	[77,78]
20	Inclusion of Social and Economic Aspects	Combining social and economic variables, as well as environmental factors, provides more comprehensive insights.	[79]
21	Awareness of Regulatory Requirements	Understanding regulatory regulations enables compliance and adherence to legal norms.	[11,47]
22	Research and Development Investment	Investing in R&D promotes innovation in LCA approaches, increasing their applicability and accuracy.	[45,54,78]
23	Verification and Validation	The dependability of LCA results is improved by independent validation and verification procedures.	[1,4,47]

### 2.4 Literature gap

A systematic literature evaluation reveals that applying LCA in manufacturing supply chains has the potential for reaching net-zero goals. However, establishing a basis for this implementation is critical. We have found various study gaps in the available literature.

The adoption of LCA has been explored in various sectors, highlighting its potential to drive sustainability and reduce carbon emissions. Kumar et al. [25] examined LCA in the transportation sector, demonstrating how it can optimize resource use and lower emissions across global supply chains. LCA plays a significant role in circular economies, focusing on waste reduction and resource efficiency in the manufacturing and construction sectors. However, research specifically addressing the adoption of LCA in the rubber industry, particularly in emerging economies like India, remains limited. Given the unique challenges faced by this industry, including high energy consumption and environmental impacts, this study seeks to fill this gap by identifying the enablers that facilitate LCA adoption. By comparing these findings with other sectors, such as automotive and textiles, we aim to provide a more comprehensive understanding of how LCA can be leveraged to achieve NZE across different industrial contexts.

1. Most research examines the numerous functions of LCA in supply chains across different manufacturing sectors, but more effort must be made to define its unique role in achieving a net-zero supply chain in the rubber industry.
2. There needs to be more relevant research to identify and compile the elements supporting LCA in the direction of a NZSC.
3. Scant research is available to assess and explain how well LCA works to achieve NZSC goals.

## 3 Research methodology

**Fig. 3:**
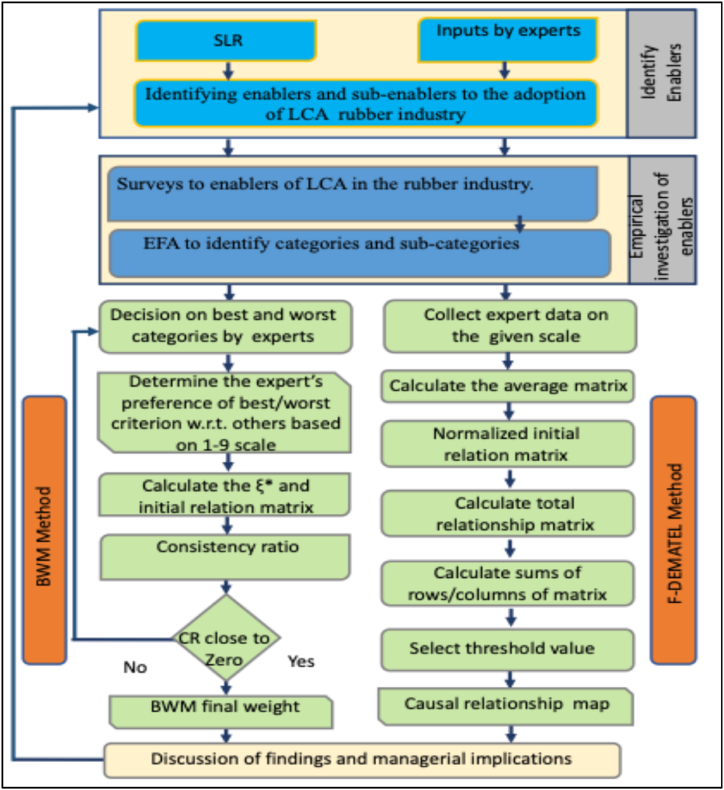
Research methodology.

The methods used in this study are shown in Fig. 3. The study establishes a theoretical foundation for the issue through an empirical examination, strengthened by qualitative and quantitative methodologies. Empirical research seeks to develop information from respondents' direct and indirect experiences. This study aims to identify and examine the enablers for LCA adoption in the rubber industry. The enablers described in the literature require validation based on respondents' perceptions of their applicability. Thus, data was gathered from a variety of manufacturing sectors in the Indian rubber industry via both online and offline data collection approaches. This empirical evaluation helps the enablers in implementing LCA. The study establishes the relative weights and cause-effect relationships between enablers and their sub-enablers. Bias checks were performed, and the data's reliability and validity were evaluated using Cronbach's alpha. Enablers were categorized appropriately using EFA.

BWM was used to determine the relative importance of the enablers [79]. This strategy is effective for resolving decision-making difficulties with various criteria. Its advantages include delivering consistent results and enabling decision-makers to identify the most effective (high effect) and least influential (low effect) criteria. According to Rezaei [80], BWM produces dependable outcomes faster since it makes fewer pairwise comparisons. Although BWM is generally accepted and utilized in many different fields, it cannot map the connections between enablers. On the other hand, because it can create links between the enablers, the Fuzzy-DEMATEL approach is more appropriate for this use case. Fuzzy-DEMATEL is a well-known technique researchers use in various domains to represent cause-and-effect interactions as a digraph.

### 3.1 Why BWM and Fuzzy-DEMATEL?

The BWM was chosen for its ability to simplify decision-making in complex environments, particularly by requiring fewer comparisons than traditional “multi-criteria decision-making (MCDM) techniques”. This makes BWM especially suitable for prioritizing enablers in a complex, multi-stakeholder environment such as the rubber industry. Fuzzy-DEMATEL, on the other hand, was used to explore causal interrelationships among these enablers. The fuzzy approach allows for the incorporation of uncertainty and imprecision in expert judgments, which is particularly valuable in the context of LCA, where different factors may interact in complex and non-linear ways. This hybrid approach provides a robust framework for both prioritizing key enablers and understanding their interdependencies, thereby offering deeper insights into LCA adoption strategies.

While BWM and fuzzy DEMATEL provided valuable insights, certain limitations were encountered during the study. BWM was the reliance on expert opinion, which may introduce subjective biases, particularly in selecting the 'best' and 'worst' enablers. While efforts were made to minimize this through a diverse panel of experts, the subjective nature of the method remains a potential limitation. The application of Fuzzy-DEMATEL posed challenges due to the complex and sometimes ambiguous relationships between enablers, especially in cases where the data was not readily available or reliable. The fuzzy aspect of DEMATEL, while useful for handling uncertainty, can lead to difficulties in clearly defining the boundaries between cause-and-effect relationships, which may affect the precision of the results.

### 3.2 Questionnaire survey development and collection of data

An empirical study was conducted to confirm the statistical reliability of all components. Based on previous research, a questionnaire was created that used a five-point Likert scale ranging from 5 (Strongly Agree) to 1 (Strongly Disagree). It was pre-tested by area experts with extensive experience in LCA from both the manufacturing sector and academia. As a result, specific questions were adjusted to improve clarity and reduce confusion. The survey was then distributed to participants in the Indian rubber and tire industries, accompanied by a cover letter emphasizing respondent confidentiality and organizational information. Initially, data were collected using a convenience sample strategy. Exchanges with participants resulted in further referrals, allowing the study team to reach more respondents. The questionnaire and cover letter were sent to 564 managers from various manufacturing industries in India. Ultimately, data from 123 respondents were collected. Following a thorough assessment, 123 (21.8 %) replies were deemed complete in all respects, indicating a sufficient sample size for analysis.

### 3.3 Empirical study

A comprehensive theoretical foundation for the study is built through an empirical investigation employing quantitative and qualitative approaches. The structure of enablers was constructed through EFA using SPSS 23.0, which allows for the study of participant data collected via a questionnaire survey.

## 4 Data analysis and results

Numerous statistical methods were used to analyze the acquired data in this study; more information is provided below. Table 3 contains information about the responses from the survey respondents.

**Table 3:** Respondent profile.

Characteristics	Sub-characteristics	Total	Percentage
Education	Graduation	72	58.53
	Post-Graduation	37	30.08
	Doctorate	14	11.38
Professional Experience	0–5 years	64	52.03
	5–10 years	45	36.58
	10–15years	9	7.31
	Above 20 years	5	4.06
Current Position	Senior manager	19	15.44
	Manager	54	43.9
	Middle Manager	15	12.19
	Supervisor	11	8.94
	Other, please specify	24	19.51
Organization Type	Small size firm	63	51.21
	Medium size firm	24	19.51
	Large size firm	9	7.31
	Educational Institutes	27	21.95

### 4.1 Biasness measurement

Several methods were used to validate the enablers and reduce the possibility of respondent biases in the opinions collected during data collection. First and foremost, the anonymity of the expert comments was guaranteed. Second, because the goals and objectives of the research were made evident to participants, they were encouraged to give the most pertinent answers. This method made capturing the respondents' most reliable responses easier, decreasing the possibility of biased answers.

### 4.2 Exploratory factor analysis

The EFA technique is commonly used in operations management to determine the factor structure of variables. It has various advantages over competing approaches, the most notable of which is the reduction of multiple variables into a more compact structure while minimizing information loss. To guarantee that data is suitable for EFA, methods such as "Bartlett's test of sphericity" and "Kaiser-Meyer-Olkin" (KMO) are widely used. Bartlett's test should provide a result of P 0.01, with a KMO value of at least 0.60. The current study's KMO score is 0.879, suggesting that the data collected from the industries is eligible for EFA analysis. Using varimax factor rotation, EFA is then used to derive the variable factor structure, as shown in Table 2, where enablers connected to LCA technologies for the rubber manufacturing industry are divided into six primary categories. This indicates a total variance of 72.94 %. The factor loading range for each challenge is 0.602 to 0.891, which exceeds the literature-recommended threshold for analysis. Table 4 shows the final results of the factor analysis for LCA enablers.

**Table 4:** EFA results for LCA enablers.

Enablers groups	Enablers	Mean	Std. deviations	Item loading	Eigen value
Data Availability and Quality (DQ)	Supplier Performance Metrics Integration (DQ1)	3.2783	0.5212	0.713	3.772
	Verification and Validation (DQ2)	3.8270	0.8376	0.786	
	Standardization of Methodologies (DQ3)	2.7823	0.5648	0.621	
	Research and Development Investment (DQ4)	3.4313	0.7089	0.874	
	Industry Standards and Best Practices (DQ5)	2.6586	0.9393	0.764	
Stakeholder Engagement (SE)	Stakeholder Engagement (SE1)	3.4749	0.9099	0.835	3.105
	Collaboration Across Supply Chain (SE2)	3.5770	0.6029	0.870	
	Public Awareness and Education (SE3)	3.2217	0.8521	0.891	
Process and Methodology (PM)	Technological Advancements (PM1)	3.8534	0.8362	0.796	1.753
	Training and Capacity Building (PM2)	3.7478	0.6736	0.743	
	Accessibility of Tools and Resources (PM3)	3.4491	0.8519	0.796	
	Flexibility in Methodological Approaches (PM4)	3.6898	0.9776	0.859	
Policy and Regulation (PR)	Regulatory Support (PR1)	3.8003	0.6153	0.772	1.414
	Policy Alignment (PR2)	3.8076	0.8831	0.774	
	Awareness of Regulatory Requirements (PR3)	3.7234	0.7726	0.637	
	Incentives for Sustainability Reporting (PR4)	3.2845	0.5707	0.602	
Organizational Integration (OI)	Life Cycle Thinking Integration (OI1)	3.6598	0.8838	0.647	1.228
	Use of Environmental Management Systems (OI2)	3.3546	0.7229	0.788	
	Continuous Improvement Culture (OI3)	2.6781	0.6047	0.780	
	Inclusion of Social and Economic Aspects (OI4)	3.5994	0.6490	0.851	
Market and Competitiveness (MC)	Transparency and Disclosure (MC1)	3.9231	0.8204	0.629	1.031
	Financial Investment (MC2)	3.4376	0.5484	0.838	
	Government Support and Funding (MC3)	3.3495	0.6469	0.848	

“Kaiser-Meyer-Olkin measure of sampling (KMO)”: 0.879; “Bartlett’s test of sphericity: Approx. Chi-Square”: 2435.268; df: 253; Sig.: 0.000. Rotation method: “Varimax with Kaiser Normalization.”

### 4.3 Ranks of enablers using BWM

Rezaei [80] proposed the BWM as a solution to decision-making problems. BWM consists of comparing selected best and worst options to other possibilities. BWM outperforms competing Multi-Criterion Decision Making approaches in terms of consistency and reliability. The steps in implementing BWM are listed below.

Step 1 To identify enablers, an in-depth review of the literature and expert perspectives are required. During this phase, information was gathered from a decision-making panel of specialists. We have one-on-one discussions with the specialists to clarify the study's objectives. The data collection approach considered details about the experts' academic backgrounds, experience levels, and employment titles.
Step 2 The results of the best-to-others preference can be deduced using Equation (1), a scale from 1 to 9 was used in the construction of the questionnaire.

(1) $$E_{B}=\left(\;e_{B1,}\;e_{B2,\ldots \ldots ,}e_{Bj}\;\right)$$

Where *E*_*Bj*_ corresponds to the importance of the best enablers over enabler *j*.

Similarly, each enabler's preference over the worst enablers is defined on a scale of 1 for the worst enablers (least effect) to 9 for the most critical. The others-to-worst preference may be found using Equation (2).

(2) $$E_{W}=\left(\;e_{W1,}\;e_{W2,}\ldots .._{,}\;e_{Wi}\right)$$

Where *E*_*Wj*_ corresponds to the importance of enabler *j* over the worst barrier *E*.

Respondents were then asked to rate each enabler based on those they considered most and least significant. The findings are presented in Table 5, Table 6.

Step 3 Calculate the weight of every enabler. To determine the optimal weights of the enabler, the maximum absolute differences {|*w*_*B*_ – *e*_*Bj*_ *w*_*j*_|, |*w*_*j*_ – *e*_*wj*_ *w*_*w*_|}, for all *j* should be minimized. The problem statement is formulated as depicted in Equation (3):

$${\mathrm{Min}\;\max}_{j}\left\{\left|w_{B}\;–\;e_{Bj}\;w_{j}\right|,\left|w_{j}\;–\;e_{wj}\;w_{w}\right|\right\}$$

**Table 5:** Best to other enablers.

Experts	Best enablers	DQ	SE	PM	PR	OI	MC
Expert 1	OI	6	9	7	8	1	5
Expert 2	SE	3	1	8	6	7	4
Expert 3	DQ	1	7	4	3	8	7
Expert 4	PM	7	4	1	5	3	6
Expert 5	PR	4	9	6	1	5	3
Expert 6	MC	8	5	3	7	3	1

**Table 6:** Worst to other enablers.

Expert	Best enablers	DQ	SE	PM	PR	OI	MC
Expert 1	PR	4	8	6	1	8	3
Expert 2	MC	7	4	7	6	5	1
Expert 3	OI	3	8	4	7	1	5
Expert 4	DQ	1	3	7	4	6	8
Expert 5	PM	7	9	1	7	5	3
Expert 6	SE	5	1	4	5	7	6

Subject to

(3) $$\sum_{j}W_{\dot{j}}=1\text{,}$$

$$W_{j}\geq 0\;\text{for}\;\text{all}\;j$$

The linear programming formulation is used to solve this problem.

Min ξ Subjected to

$$\left|w_{B}\;–\;e_{Bj}\;w_{j}\right|\leq ,\text{for}\;\text{all}\;j$$

$$\left|w_{j}\;–\;e_{wj}\;w_{w}\right|\leq ,\text{for}\;\text{all}\;j$$

(4) $$\sum_{j}W_{\dot{j}}=1$$

$$W_{j}\geq \text{for}\;\text{all}\;j$$

Equation (4) should have a unique solution.

The optimal weights (*w*_*1*_∗,*w*_*2*_∗_,. …,_ *w*_*n*_∗) are listed after solving the LPP (Linear Programming Problem). To measure the consistency of the responses, Equation (5) is used:

(5) $$\text{Consistency}\;\text{ratio}\;\left(\text{CR}\right)=\left(\xi^{*}\right)/\left(Consistency\;Index\;\left(CI\right)\right)$$

The lower the ‘consistency ratio’, the higher the comparisons' reliability.

The consistency of each expert matrix is obtained using the above equation (5), where all values are near zero. After completing all of the BWM technique's processes, each enabler's weight is calculated for each expert's matrix. Table 7 shows the final weights and rank. Each sub-enabler and its main enabler weight are computed using a similar process, and the global rank is displayed.

**Table 7:** Ranking of enablers to LCA.

Enablers	Relative weight	Sub enablers	Relative weight	Local rank	Global weight	Global rank
Data Availability and Quality (DQ)	0.173	DQ1	0.1636	3	0.0800	7
		DQ2	0.1947	2	0.0350	20
		DQ3	0.1132	5	0.0567	11
		DQ4	0.1528	4	0.0524	13
		DQ5	0.2651	1	0.0465	17
Stakeholder Engagement (SE)	0.097	SE1	0.1739	3	0.0988	1
		SE2	0.2615	2	0.0391	18
		SE3	0.1639	4	0.0811	5
Process and Methodology (PM)	0.082	PM1	0.3998	1	0.0765	9
		PM2	0.3199	1	0.0570	10
		PM3	0.2760	2	0.0143	22
		PM4	0.0916	3	0.0190	21
Policy and Regulation (PR)	0.164	PR1	0.1712	4	0.0827	4
		PR2	0.3356	2	0.0947	2
		PR3	0.3263	3	0.0548	12
		PR4	0.3658	1	0.0795	8
Organizational Integration (OI)	0.253	OI1	0.1570	4	0.0897	3
		OI2	0.2477	2	0.0117	23
		OI3	0.2191	3	0.0489	16
		OI4	0.3604	1	0.0804	6
Market and Competitiveness (MC)	0.155	MC1	0.2072	1	0.0518	14
		MC2	0.1811	2	0.0502	15
		MC3	0.1016	3	0.0363	19

### 4.4 Fuzzy-DEMATEL approach for causal relationship between enablers

The Fuzzy-DEMATEL method provides a methodical way to determine and evaluate the causal relationships between multiple complex factors. It helps to highlight the degree of interaction among these criteria and the direct relationship between them. Particular academics have proposed that Fuzzy-DEMATEL is a good fit for assessing the overall effect intensity across the requirements and identifying direct and indirect causal linkages. Furthermore, the Fuzzy-DEMATEL approach skillfully handles the interdependencies among the various criteria. Below is further information about the computing process of the Fuzzy-DEMATEL approach.

The first step in the Fuzzy-DEMATEL approach is to create a direct relation matrix, which is a relationship matrix that is produced by gathering data from experts. After that, the raw data is converted into fuzzy sets, which gives the input relation matrix a triangular fuzzy set. A single direct relationship matrix is created by combining the unique relationship matrices that each expert gave. The average direct relation matrix is then obtained by translating this fuzzy average matrix into precise integers.

Step 1 To measure the degree of relationship between two dimensions, we first create a *n* × *n* matrix to produce the direct relationship matrix. Table 8 shows how this matrix uses a triangular fuzzy number. The direct interactions between dimensions are represented by this matrix, which we will refer to as X. More specifically, dimension *i* affects dimension *j* in this situation.

**Table 8:** Fuzzy scale.

Response	Linguistic terms	Linguistic terms and triangular fuzzy numbers
1	Very important	(0.9, 0.05, 0.05)
2	Important	(0.75, 0.2, 0.05)
3	Medium	(0.5, 0.4, 0.1)
4	Unimportant	(0.25, 0.6, 0.15)
5	Very unimportant	(0.1, 0.8, 0.1)

$$X={\left[X_{\text{Ij}}\right]}_{n*n}$$

**Step 2:** Normalization

$${\text{XL}}_{\text{Ij}}^{k}={{a_{1}}^{k}}_{\text{ij}}\;–\min\left({{a_{1}}^{k}}_{\text{ij}}\right)/\left[\max\left({{a_{n}}^{k}}_{\text{Ij}}\right)-\min\left({{a_{n}}^{k}}_{\text{Ij}}\right)\right]$$

XMijk=a2kij–min(a2kij)/[max(ank)Ij−min(ankIj)]

XkIj=a3kij–min(a3kij)/[max(ank)Ij−min(ankIj)]

Step 3 Computing the left (XLS ^k^_Ij_) and the right (XRS ^k^_I j_) normalized values.

XLSkIj={XMkIj/(1+XMkIj–XLkIj)}

XRSkIj={XRkIj/(1+XRkIj–XMkIj)}

Step 4 Calculation of crisp values

XkIj={[XLSkij∗(1−XLSkIj)–XRSkIj∗XRSkIj]/(1–XLSkIj+XRSkIj)}

Step 5 Generating normalized crisp values

ZkIj=min(ankIj)+Xk∗Ij[max(ank)Ij−min(ank)Ij]

Step 6 Calculate the Direct relation matrix and the sum of rows. This is done by taking an average of all normalized crisp values of different experts and using it to form a direct relation matrix. Table 9 shows the average matrix, Table 10 shows the Normalized direct relation matrix, and Table 11 shows the total relation matrix (T) of the data collected for Fuzzy- DEMATEL.

**Table 9:** Average matrix.

Enablers	DQ	SE	PM	PR	OI	MC	SUM
DQ	0.8610	0.2493	0.2829	0.2557	0.2967	0.2690	2.2146
SE	0.2464	0.8062	0.2568	0.2351	0.2751	0.2798	2.0995
PM	0.2649	0.2185	0.8287	0.2921	0.2921	0.2470	2.1432
PR	0.2763	0.1869	0.2360	0.8837	0.2833	0.2155	2.0817
OI	0.1921	0.2401	0.2067	0.2151	0.8588	0.2365	1.9493
MC	0.2255	0.2549	0.2155	0.2136	0.2457	0.8956	2.0508

**Table 10:** Normalized direct relation matrix (d).

Enablers	DQ	SE	PM	PR	OI	MC
DQ	0.3888	0.1126	0.1278	0.1154	0.1340	0.1215
SE	0.1113	0.3640	0.1159	0.1061	0.1242	0.1264
PM	0.1196	0.0987	0.3742	0.1319	0.1319	0.1115
PR	0.1248	0.0844	0.1066	0.3990	0.1279	0.0973
OI	0.0867	0.1084	0.0933	0.0971	0.3878	0.1068
MC	0.1018	0.1151	0.0973	0.0965	0.1109	0.4044

**Table 11:** Total relation matrix (T).

Enablers	DQ	SE	PM	PR	OI	MC	Enablers
DQ	3.1905	2.6333	2.7693	2.8699	3.1863	2.9721	14.6493
SE	2.6198	2.7954	2.5651	2.6615	2.9568	2.7849	13.5986
PM	2.7062	2.4980	2.9818	2.7822	3.0509	2.8291	14.0191
PR	2.6213	2.3826	2.5242	3.0505	2.9367	2.7011	13.5153
OI	2.3261	2.2212	2.2821	2.3914	3.0352	2.4873	12.2561
MC	2.5226	2.3914	2.4536	2.5611	2.8419	3.0884	12.7706
Ci	13.4638	12.5304	13.1226	13.7556	15.1659	13.7746	
Threshold alpha value (α)	2.7131

Table 10 shows the normalized direct relationship matrix generated using expert responses' mean values.

T = D(I-D)^^−1^

We must compute the internal relation matrix's threshold value to get the alpha. The network relationship map produced by this computation, which ignores partial interactions inside the matrix. The map only displays relationships whose values are more significant than the threshold; relationships whose values are less than the threshold are considered zero. The threshold value in this instance is 2.7131.

Following the creation of the average crisp direct relation matrix, the subsequent step involves developing a normalized relation matrix. A total relation matrix is utilized to classify drivers into cause-and-effect groups. Each row and column of this matrix is summed up and denoted as ‘Ri’ and ‘Ci’ respectively. The values of (Ri + Ci) and (Ri − Ci) are calculated, where (Ri + Ci) indicates the significance of a driver based on its reliance on others, and (Ri − Ci) represents the driver's impact on the system. Positive (Ri − Ci) values are classified as cause group drivers, while negative values indicate effect group drivers.

**Fig. 4:**
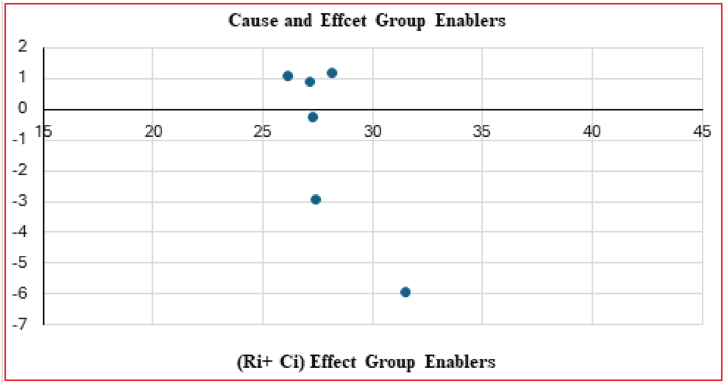
Causal relationship diagram for LCA enablers.

In the Fuzzy-DEMATEL approach, negative Ri-Ci values in Table 12 represent the effect of factors, whereas positive Ri-Ci values propose causal factors. As a result, PR, OI, and MC are considered causal factors, whereas DQ, SE, and PM are regarded as effects. Fig. 4 explains the Causal relationship diagram for LCA enablers.

**Table 12:** Importance relation.

	Ri	Ci	Importance	Relation	T i	Weights
			Ri + Ci	Ri-Ci	(Ri + Ci)/2	T i/Average Of T i
DQ	14.6493	13.4638	28.1131	1.1855	14.0565	0.1678
SE	13.5986	12.5304	26.1290	1.0682	13.0645	0.1560
PM	14.0191	13.1226	27.1417	0.8965	13.5708	0.1620
PR	13.5153	13.7556	27.2709	−0.2403	13.6355	0.1628
OI	12.2561	15.1659	27.4220	−2.9099	13.7110	0.1637
MC	12.7706	18.6995	31.4702	−5.9289	15.7351	0.1878

**Fig. 5:**
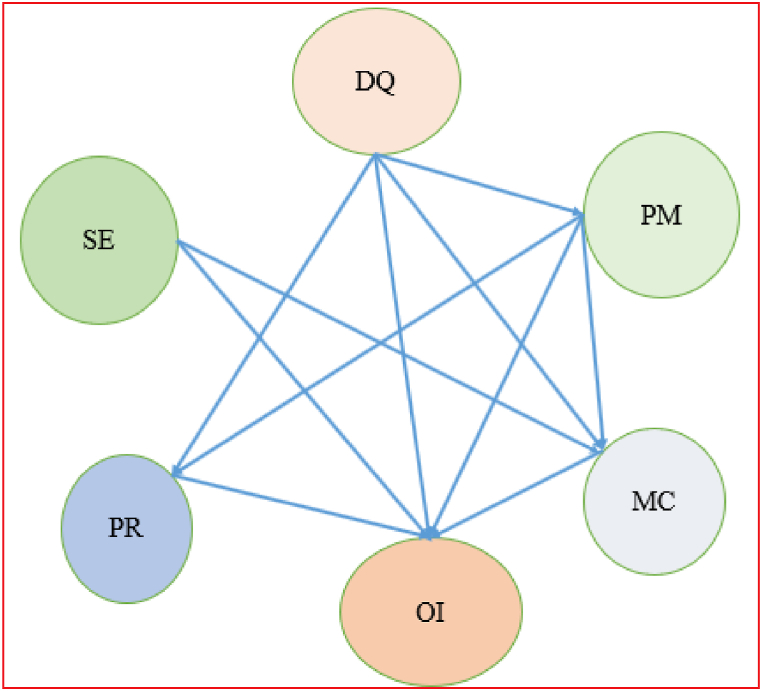
Network map of main group enablers (Authors' work).

In Fig. 5, a network map of the main group enablers, we can observe the effect of all the enablers on each other. We have used Table 12 to find how one enabler impacts the other enablers by observing the elements of the total relationship matrix having values more significant than the threshold (alpha) value.

## 5 Discussion

This study uses SLR to identify key enablers of LCA adoption in the rubber and tire manufacturing industry, each contributing to the broader goal of achieving an NZSC. The results of the BWM revealed that “Organizational Integration” was the most significant enabler, with a relative weight of 0.253. “Data Availability and Quality” followed closely, reflecting its importance in facilitating effective decision-making for LCA adoption, highlighting the importance of internal collaboration and alignment across departments such as logistics, production, and procurement. “Stakeholder Engagement”, both internal and external, was also identified as an important enabler, emphasizing the need for collaboration across the SC. Engaging suppliers, customers, and regulators is key to creating a circular economy, as it enables the sharing of best practices and the alignment of goals. “Policy and Regulation” were found to play a crucial role in driving LCA adoption, particularly in emerging economies, where government incentives and regulations can accelerate the shift toward sustainable practices. These enablers not only support the adoption of LCA but also contribute to building more resilient, sustainable SCin the rubber and tire manufacturing industry and beyond. Fuzzy DEMATEL further highlighted the causal relationships between enablers, with “Policy and Regulation” being the most influential causal factor, affecting other enablers such as “Stakeholder Engagement” and “Process and Methodology”. These findings provide a clear ranking of enablers and offer insights into how they interact to drive LCA adoption in the rubber and tire manufacturing industry.

The findings of this study are consistent with previous research in other industries [47]. also identified “Organizational Integration” as a key enabler in their study on sustainable SC. However, our study extends these findings by highlighting the importance of “Data Availability and Quality”, particularly in the context of LCA adoption in the rubber and tire manufacturing industry. The causal interrelationships identified through fuzzy DEMATEL suggest that “Policy and Regulation” are crucial in shaping the broader landscape for sustainability efforts. These results indicate that external pressures, such as regulatory frameworks and market incentives, can significantly influence internal organizational practices, particularly in emerging economies.

## 6 Implications

This study focuses specifically on the rubber and tire manufacturing industry in India, an emerging economy with significant environmental challenges. The rubber industry is a major contributor to GHG emissions, and its transition toward net-zero practices is crucial for achieving the country’s broader sustainability goals. The findings of this study, however, are not limited to the rubber industry. The identified enablers, such as Organizational Integration, Data Availability, and Stakeholder Engagement, apply to other manufacturing sectors, particularly those in emerging economies that face similar challenges in adopting LCA. These sectors include automotive, textiles, and chemicals, with complex SC and significant environmental impacts. The insights gained from this study can help guide these industries in their efforts to implement LCA and transition to more sustainable business models.

### 6.1 Theoretical contribution

This study contributes to the growing body of literature on sustainability and SC by focusing on adopting LCA in the rubber industry, a sector with significant environmental impact. The integration of LCA with the broader goal of achieving NZE enriches theoretical discussions on the role of circular economy principles within traditional manufacturing industries. By identifying key enablers such as Policy and Regulation, Organizational Integration, and Stakeholder Engagement, the study provides an understanding of how LCA can act as a strategic tool for sustainable operations [33].

This study offers key managerial implications for the rubber industry and related sectors pursuing sustainability through LCA. Managers should prioritize aligning with evolving regulatory frameworks to ensure compliance and enhance brand reputation. Investing in advanced technologies, such as LCA tools and digital tracking systems, is essential for improving SC transparency and resource efficiency. Strong collaboration with suppliers and stakeholders is also crucial for overcoming implementation challenges and driving innovation. Managers should view LCA not just as a regulatory requirement but as a strategic tool to gain a competitive edge by meeting the growing market demand for sustainable products and practices.

### 6.2 Practical contribution

From a practical standpoint, this study offers actionable insights for decision-makers in the rubber industry and other resource-intensive industries. The identified enablers, such as Policy and Regulation and Data Availability and Quality, can guide industries toward more efficient LCA implementation, helping them reduce their environmental footprint while improving operational efficiency. This study underscores the importance of aligning corporate strategies with evolving sustainability policies by highlighting the role of government regulations and industry standards. The practical insights can assist companies in adopting circular economy practices by identifying key areas within their SC for improvement. The research also stresses the importance of collaboration between stakeholders, suppliers, manufacturers, and policymakers in driving LCA adoption, making it a crucial tool for industries aiming to achieve NZE.

## 7 Conclusions

This study has identified potential enablers through SLR for adopting LCA within the rubber industry, focusing on achieving NZE in SC. By employing a hybrid approach using the Best-Worst Method and fuzzy DEMATEL, key enablers such as organizational integration, data availability and quality, stakeholder engagement, technological capability, and regulatory frameworks were identified and prioritized. These findings offer valuable insights for industry stakeholders looking to implement LCA as a strategic tool for sustainability. The study highlights the crucial role of collaboration between internal departments and external stakeholders, underscoring the role of government regulations and policy frameworks in promoting LCA adoption. Regulatory incentives, such as emissions reduction targets and waste management policies, can accelerate the shift towards more sustainable SC.

Technological advancements, such as recycling innovations and digital tracking systems, also offer significant opportunities for optimizing resource use and minimizing waste, further enabling circular economy practices within the rubber industry. While this study focuses on the rubber industry in India, the enablers identified are relevant to a wide range of industries with similar SC structures. Industries such as automotive, textiles, and agriculture, which also rely on complex, resource-intensive SC, can benefit from the insights provided in this study. The emphasis on Organizational Integration, Data Quality, and Policy Support applies broadly to any industry looking to adopt sustainable practices through LCA. The findings apply to other emerging economies where regulatory pressures and market demands are increasingly driving the adoption of LCA. By identifying and prioritizing key enablers, this study offers a framework that can be adapted to various industrial contexts, helping organizations in different sectors transition toward NZSC performance.

### 7.1 Limitations and future research scope

This study, while offering valuable insights into the adoption of LCA for NZE in the rubber industry, has many limitations. The research is industry-specific, focusing solely on the rubber and tire manufacturing sector, which may limit the generalizability of the findings to other industries. Although the identified enablers might apply to similar sectors, further validation across industries such as textiles or plastics is needed. The study relies heavily on qualitative data such as expert opinion and case studies, and while these offer rich insights, including quantitative data through surveys or statistical models would strengthen the validity of the results. The study takes a present-day view and does not account for long-term shifts in regulations, market dynamics, or technological advancements that could influence LCA adoption in the future.

For future research, cross-industry studies could reveal whether the identified enablers are consistent across different sectors. Expanding the geographical scope would provide a global perspective on LCA adoption. Incorporating quantitative methods, such as structural equation modelling, could validate the findings. Exploring the role of emerging technologies like AI and blockchain in enhancing LCA would offer valuable insights and enrich the understanding of LCA’s role in driving sustainability.

## CRediT authorship contribution statement

**Alok Yadav:** Visualization, Methodology, Investigation, Conceptualization. **Anish Sachdeva:** Software, Project administration, Data curation. **Rajiv Kumar Garg:** Methodology, Investigation, Data curation, Conceptualization. **Karishma M. Qureshi:** Validation, Methodology, Formal analysis, Data curation. **Bhavesh G. Mewada:** Investigation, Formal analysis, Data curation, Conceptualization. **Naif Almakayeel:** Investigation, Formal analysis, Data curation, Conceptualization. **Mohamed Rafik Noor Mohamed Qureshi:** Writing – review & editing, Writing – original draft, Funding acquisition, Conceptualization.

## Informed consent statement

Not Applicable.

## Institutional review board statement

Not Applicable.

## Data availability statement

Data will be made available to researchers at their request.

## Funding

This research was funded by the 10.13039/501100023674Deanship of Scientific Research, King Khalid University, Kingdom of Saudi Arabia, and the large grant number is RGP. 2/476/44.

## Declaration of competing interest

The authors declare that they have no known competing financial interests or personal relationships that could have appeared to influence the work reported in this paper.
